# 

**Published:** 1839

**Authors:** 


### MEDICO-CHIRURGICAL REVIEW,

AND

### JOURNAL

OF

### PRACTICAL MEDICINE.

*(NEW SERIES.)*

VOLUME THIRTY-ONE,

[1st of APRIL to 30th of SEPTEMBER,]

1839.

VOL. XI. of DECENNIAL SERIES.

EDITED

BY JAMES JOHNSON, M. D.

PHYSICIAN EXTRAORDINARY TO THE LATE KING,

AND

HENRY JAMES JOHNSON, ESQ.

LECTURER ON ANATOMY AT THE SCHOOL OF ST. GEORGE'S HOSPITAL IN KINNERTON STREET.

Surgeon Genl's Office LIBRARY. 57471 Washington, D.C.

Brown

LONDON:

PUBLISHED BY S. HIGHLEY, 32, FLEET STREET,

*Re-printed in New York, by Mr. Wood.*

1839.

WI
ME75T

PRINTED BY F. HAYDEN,
Little College Street, Westminster.

### INDEX.

A.

Aberdeen Lunatic Asylum, report of.. 594

Abdomen, encysted dropsy of the.... 541

Abdominal tumors, Dr. Bright on.... 256

Abernethiana .................... 325

Abernethy's courtship ............ 325

Abscess in the iliac region.......... 239

Absence of the uterus.............. 547

Abstracts of cases by Sir D. Dickson.. 607

Abuse of purgative medicines...... 86

Abuses of medical charities.......... 357

Aconite, cases of poisoning by ...... 544

Actual cautery, utility of the........ 276

Addison on cerebro-spinal affections.. 241

Administration of mercury ........ 484

Advantages of a speculum-cushion .. 297

Ætiology of club-feet............... 200

Affections of the brain, bleeding in .. 81

Affections of the bones, venereal .... 483

Ague, arsenic given for ............ 390

Aitk[illegible]s on the fucus esculentus as a [illegible]m bougie.................... 48

Alb[illegible]inous urine ................ 25

Alc[illegible]n surgical injuries.......... 488

Alis[illegible] atmospheric contagion .... 93

Alk[illegible] or lithic acid gravel........ 38

All[illegible] on the classification of the insane 60

Alison's experiments on venous pulsation ...................... 189

Alternating depositions of lithic acid and phosphatic calculi.......... 39

Alum, internally, in gonorrhœa.. ... 216

Amaurosis, treatment of .......... 219

Amenorrhœa, treatment of ........ 222

America, progress of medicine in .... 584

Amputations .................... 578

Analysis of bones affected with mollities ossium .................... 246

Anatomy, studentships in ... ...... 284

Andral on the coagulation of the blood 231

Aneurysm of the pulmonary artery .. 149

Annual report of the Registrar-general 443

Antimonial medicines.............. 92

Antiquity of vaccination .......... 286

Anus, fissures of the .............. 529

Aphthous inflammation of conjunctiva 68

Application of cold in fevers........ 394

Applications in ophthalmia ........ 273

Arm, case of paralysis of the........ 220

Arnott on diseases of the eye ...... 280

Arrested development, Cruveilhier on, 201

Arsenic given for ague ............ 390

Art of rising in physic ............ 310

Arteries, Kirkbride on wounds of.... 184

Arthritic iritis.................... 77

Ascites.......................... 541

Ashwell on incision of the uterus.... 253

Atmospheric contagion, Alison on.... 93

Attention, Dr. Holland on........... 181

Auscultation of the heart, M. Beau on 535

Autoplasty after excision of cancerous tumors ...................... 235

B.

Baldness, Dupuytren's pommade for.. 240

Baltimore alms-house hospital report 273

Barataria.......................... 179

Barren mammæ, case of prolific uterus with.......................... 150

Barron, Mr. and Mr. Pettigrew...... 296

Bartlett on a chronic cerebral affection 566

Beau on the auscultation of the heart 535

Belladonna, utility of, in epilepsy .... 543

Bermudas ...................... 460

Bermudas, diseases of the lungs in the 462

Bermudas, diseases of the stomach and bowels in ...................... 462

Bimeconate of morphia............. 190

Biographical memoir of Dr. Heberden 122

Biographical memoir of Dr. Cheyne.. 129

Birmingham School of Medicine report 608

Births, registration of ............ 445

Bitters.......................... 418

Bladder, secale cornutum in paralysis of the ........................ 221

Blandin on traumatic paralysis...... 220

Bleeding in affections of the brain.. . 81

Blistering the eyelids, ophthalmia treated by ........................ 562

Blood, changes in the, in inflammation 229

Blood, coagulation of, in the vessels.. 231

Blood globule, description of the. .. 352

Blood, coagulation of the .......... 374

Blood, condition of the, in the living vessels ...................... 507

Blundell on midwifery .......... 177

Blushing, Mr. Burgess on the mechanism of ...................... 173

Blushing, poetry of................ 173

Bombay medical society, transactions of the ........................ 384

Bones, venereal affections of the .... 483

Botany, British, Dr. Macreight's .... 179

Botany for schools, by Lindley ..... 179

Bouillaud's eloge on Broussais ...... 195

Bowels, obstruction of the ........ 576

Brachial artery, bayonet wounds of the 185

Brain, bleeding in affections of the .. 81

Branchial apertures in the decidua .. 520

Breeding leeches, mode of.......... 290

Bright on abdominal tumors........ 256

British America, mortality of troops in 460
Brodie on vertebral diseases ........ 277
Brodie's treatment of gouty acidity . 279
Broussais, eloge on................ 195
Broussais, last illness of............ 197
Broussais, anecdote of ........... 518
Broussaisism, decline of, in France .. 517
Bruit de soufflet, mechanism of the.. 575
Bubo ......................... 482
Burgess on the mechanism of blushing 173
Buxton, Sir C. Scudamore on springs of 162
Buxton waters, analysis of the...... 162
Buxton waters, effects of the........ 163
C.
Cæsarian operation .............. 548
Cæsarian operation, successful case of 549
Canada ......................... 467
Canada, condition of the hospitals in 468
Canada, diseases of................ 469
Cancer.......................... 355
Cancer of the mamma, case of...... 235
Cancer of the lower lip ............ 236
Cancer of the tongue............... 267
Cancerous tumors, autoplasty after excision of ..................... 235
Carbuncle, treatment of, with caustic alkali ........................ 149
Carcinoma of the hepatic ducts, Dr. Hake on ....................... 348
Carcinoma, varicose capillaries the cause of ....................... 351
Carpenter on physiology .......... 165
Carpenter's inaugural dissertation.... 497
Case of the Rev. F. Jardine ........ 424
Cataract, Indian operation for ...... 388
Catarrhal ophthalmia............... 66
Catarrhal ophthalmia, treatment of.. 66
Catarrhus senilis ................. 402
Caustic alkali, treatment of carbuncle with.......................... 149
Caustic, treatment of nævi with .... 227
Cerebral affections, general character of .......................... 242
Cerebral affections connected with renal disease .................. 242
Cerebro-spinal affections, Dr. Addison on ......................... 241
Cervix uteri, occlusion of the ...... 254
Chancres........................ 481
Chancres, Hunter's definition of .... 481
Changes of the blood in inflammatory diseases ..................... 229
Changes which occur in old age .... 400
Chemosis of the corneal conjunctiva 75
Cheyne, Dr. biographical memoir of.. 129
Chilblains, useful application to .... 227
Chronic bronchitis, copaiba balsam in 226
Chronic cerebral affection......... 566
Chylous urine.................... 25
Circular to medical practitioners .... 448
Circular and flap amputations ...... 579
Civilization, influence of, on insanity 551
Classification of the insane ........ 60
Climate of Guiana................ 52
Club-feet, ætiology of ............ 200
Club-foot, case of ............... 524
Coagulation of blood in the vessels .. 231
Colchicum in gout................ 87
Colchicum, external use of ........ 190
Colica pictonum, observations on .. . 151
Colica pictonum, treatment of...... 152
Colon, pathology of the........... 403
Condition of the hospitals in Canada 468
Conjunctiva, pustular inflammation of the ......................... 68
Conjunctiva, variolous inflammation of the ....................... 70
Consumption, deaths from ........ 454
Cooper's treatment of varicocele .... 247
Copaiba balsam in chronic bronchitis 226
Corneal conjunctiva, chemosis of the 75
Corpuscules, size of the .......... 354
Corrigan on the mechanism of the bruit de soufflet ................ 575
Cowan on medical quackery ....... 511
Creosote, styptic action of ........ 225
Cruveilhier on arrested development 201
D.
Danger of resuscitating a jail bird .. 333
Danger of medical quackery, Dr. Cowan on ........................ 511
Davies' selections in pathology and surgery......................... 377
Death of Talma ................. 233
Death of Shakspeare .............. 501
Death from hanging, signs of ...... 550
Deaths of doctors .............. .. 332
Deaths at particular ages .......... 446
Deaths from small-pox ............ 453
Deaths from mental emotions ...... 454
Deaths from consumption.......... 454
Deaths from intemperance and starvation ........................ 455
Decandolle's vegetable organography 179
Deccan, mode of breeding leeches in 390
Decidua, branchial apertures in the .. 521
Decline of Broussaisism in France .. 517
Deficiency of food, Dr. Howard on the effects of ..................... 509
Delirium tremens, treatment of .... 572
Delirium tremens, Dr. Wolff on .... 499
Description of the pus globule ...... 352
Description of the blood globule .... 352
Detection of the salts of lead, Orfila on 208
Dezeimeris on re-vaccination ...... 193
Dezeimeris on ruptures of the heart.. 531
Diabetes mellitus ................ 34
Diabetes, relative quantity of ingesta and egesta in ................. 35
Diabetes, proximate cause of ........ 35
Diabetes, treatment of............ 36
Diabetic urine, existence of urea in .. 36

Diagnosis of vertebral disease ...... 277

Diagnostic marks of syphilitic iritis .. 76

Diary of the Rev. Mr. Ward........ 501

Dick on porrigo .................. 502

Diet.............................. 414

Digestion, disorders of ............ 414

Digestive organs, diseases of the .... 454

Digitalis, use of .................. 90

Digitalis, improved tincture of ...... 183

Dilatation of the os uteri in lingering labour ....................... 43

Diluents, use of ................. 85

Disease, influence of weather on .... 393

Diseased hepatic ducts, matter contained in ..................... 352

Diseases of the urinary organs ...... 25

Diseases of women, Jones on the . .. 53

Diseases of the eye, Morgan on the .. 64

Diseases of the knee-joint, Mr. Merryon on ........................ 299

Diseases of the digestive organs...... 454

Diseases of the urinary organs ...... 454

Diseases of the tegumentary system.. 454

Diseases of the town and country .. 455

Diseases of Canada................ 469

Dislocation of the head of the humerus 579

Dislocation of the upper end of the radius ......................... 580

Disorders of digestion ............ 414

Diurnal variations of the pulse...... 244

Division of the tibia.. ............ 249

Doctrine of arrested development.... 201

Donovan's improved tincture of digitalis ......................... 183

Dropsies, Dr. Osborn on .......... 295

Dropsy and consumption curable.... 513

Dublin cow-pox institution, report of the ........................... 188

Dublin University, new regulations .. 590

Dubois, malpraxis of .............. 548

Dundee Royal Asylum, report of the.. 591

Dupuytren's pommade for baldness .. 240

Duration of pregnancy ............ 424

Dysentery, use of sugar of lead in.... 150

Dysentery, treatment of..... ...... 274

Dyspepsia, are vegetables injurious in 416

E.

Eccentric medical men ............ 320

Ectropeon ........................ 79

Effects of deficiency of food ..... .. 509

Elaterium for hypertrophy of the heart 277

Electricity as a medical agent........ 396

Electro-puncture in paralysis ...... 220

Elliotson's principles of medicine.... 366

Eloge on Broussais...... ........ 195

Emetics, use of ................... 89

Eminent medical men ............ 327

Encysted dropsy of the abdomen.... 541

Eneuresis......................... 560

Endocarditis, M. Legroux on ...... 209

Endocarditis in acute rheumatism.... 276

Enlargement of the kidney ........ 257

Entropeon .. ...................... 79

Epilepsy, utility of belladonna in . .. 543

Ergot, Mr. Rose on the use of the .. 43

Ergot of rye in paralysis .......... 222

Erysipelas ....................... 265

Erysipelas ....................... 380

Esquirol and Marc on insanity ... .. 206

Evils of medical charities .......... 357

Examination of an inflamed eye .... 67

Expectation of life at various ages.... 445

Experiments on the poison of the rattle-snake...................... 155

Experiments on the Wourali poison.. 185

Experiments on venous pulsation.... 189

Experiments on living animals ...... 505

Eye, Morgan on the diseases of the .. 64

Eye, treatment of diseases of the .... 65

Eye, Arnott on diseases of the ...... 280

Eyelids, application of the nitrate of silver to the..................... 75

External use of colchicum.......... 190

F.

Fatal rupture of the spleen ........ 391
Femoral artery, puncture of the .. . 184
Fenner on the advantages of a speculum cushion.................... 297
Fever ........................... 477
Fevers, liability of soldiers to ...... 17
Fevers, application of cold in ...... 394
Fevers in the Bermudas ............ 462
Fevers in Upper Canada............ 469
Fibrous structure, tendency of, to disease ........................... 483
Fissure of the rectum.............. 265
Fissures of the anus, M. Jourbert on 529
Flourens on the structure of the mucous membrane ................ 527
Food ............................ 414
Food and sleep, relations of ........ 415
Foramen ovale, case of patency of the 211
Forensic medicine ................ 424
Formation and growth of urinary calculi ........................... 38
Fothergill, anecdotes of............ 330
Fowler's case of molluscum ........ 47
Fox on intus-susceptio .......... .. 46
Fractures of the thigh ............ 263
Fractures of extremities, treatment of, 589
France, decline of Broussaisism in.... 517
Friederich on the internal use of alum in gonorrhœa .................... 216
Fucus esculentus, as a rectum bougie 48
Furnivall on phthisis .............. 289

G.

Gangrenous inflammation of the mouth 560
General and comparative physiology, Mr. Carpenter on ............... 165
Gibraltar, medical statistics of ...... 11
Gibraltar, yellow fever in .......... 12
Glanders in the human subject...... 233

Glans penis, bite of a spider on the.. 565
Gonorrhœa, M. Ricord on.......... 215
Gonorrhœa, internal use of alum in.. 216
Gonorrhœa, use of parsley in ...... 221
Gonorrhœa, identity of, with syphilis, 480
Gout, use of colchicum in.......... 87
Gout, Harvey's treatment of the .... 335
Gout ........................ 382
Gouty acidity, treatment of ........ 279
Growth of urinary calculi.......... 38
Guerin on lateral deviations of the spine ........................ 523
Guiana, climate of ................ 52
Guy on the diurnal variations of the pulse ........................ 244
Guy's Hospital reports ............ 241

H.

Hæmaturia .................... 28
Hæmaturia, causes of.............. 29
Hæmaturia, treatment of .......... 30
Hake on carcinoma of the hepatic ducts 348
Halliday on the West Indies ........ 49
Hanging, signs of death from ...... 550
Harlan on colica pictonum ........ 151
Harlan's medical & physical researches 149
Hare-lip ........................ 265
Harvey's treatment of the gout...... 335
Head of the humerus, dislocation of 579
Heart, M. Dezeimeris on ruptures of 531
Heart, M. Beau on the auscultation of 535
Heart, cause of the impulse of the .. 536
Heberden, Dr. biographical memoir of 122
Hepatic ducts, Hake on carcinoma of 348
Hereditary disease ................ 406
Hernia cerebri, Mr. Mallet on ...... 45
Hernial sac, inflammation of the.... 268
Hernia cerebri, treatment of........ 46
Hints on intermittent neuralgia...... 545
Hip, injuries and diseases of the .... 237
Hip-joint, scrofulous disease of the.. 279
History of British birds, Yarrell's.... 178
Holland's medical notes and reflections 81
Holland on attention .............. 181
Holland's medical notes and reflections 392
Hospitals in Canada .............. 468
How to get a practice ............ 310
Howard on the effects of deficiency of food ........................ 509
Human subject, case of glanders in the 223
Hydriodate of potash in syphilis .... 539
Hydrocele, tincture of iodine for .... 191
Hydrocele ...................... 265
Hydrocele cured.................. 270
Hydrocele, injection for............ 277
Hypertrophy of the os uteri, Dr. Kennedy on ...................... 159
Hypertrophy of heart, elaterium for.. 276
Hypothesis of morbid growths...... 355

I.

Identity of gonorrhœa with syphilis .. 480
Iliac region, case of abscess in the .... 239
Illustrations of medical quackery.... 317
Imperforate uterus, case of ........ 251
Impotence, a consequence of diabetes 35
Improved tincture of digitalis ...... 183
Inaugural dissertation, Mr. Carpenter's 497
Incision of the uterus, Dr. Ashwell on 253
Incontinence of urine, Dr. Lendrick on 567
Indian lithotomist ................ 387
Indian operation for cataract........ 387
Indian physician, his studies........ 389
Infants, purulent ophthalmia of...... 73
Inflammation of the conjunctiva .... 68
Inflammation of the hernial sac .... 268
Inflammation of the joints.......... 381
Inflammation of the mammæ ...... 382
Inflammation of the prostate, nitrate of silver for...................... 580
Inflammatory diseases, changes of the blood in .................... 229
Influence of physical agents on life .. 385
Influence of the weather .......... 393
Influence of civilization on insanity.. 551
Influenza, mortality from .......... 452
Ingesta and egesta, relative quantities of, in diabetes.................. 35
Injection for hydrocele ............ 278
Injuries and diseases of the hip...... 237
Insane, Allen on the classification of the 60
Insanity, Esquirol and Marc on...... 206
Insanity, influence of civilization on. 550
Intemperance and starvation, deaths from........................ 455
Intermittent neuralgia, hints on...... 545
Internal piles, M. Joubert on........ 529
Internal administration of iron ...... 226
Intus-susceptio, Mr. Fox on........ 46
Intus-susception, remarkable case of 154
Iodic acid, decomposition of, by uric acid ........................ 574
Iodine and mercury, compound salts of, in syphilis .................... 216
Iodine, powers and applications of .. 377
Iodine, mode of exhibiting.......... 379
Ionian Islands, medical statistics of the 20
Iris, natural coloration of the ...... 75
Iritis, use of turpentine in.......... 280
Iritis, syphilitic, diagnostic marks of.. 76
Iritis, Dr. M'Cabe on.............. 597

J.

Jail-bird, danger of resuscitating a .. 333
Jardine, the Rev. Fergus, case of .... 424
Joints, inflammation of the ........ 382
Jones on the diseases of women .... 53
Joubert on internal piles............ 529

K.

Kennedy on hypertrophy of the os uteri 159
Kennedy on occlusion of the vagina and uterus .................... 571
Key's case of division of the tibia .. 249
Kidney, enlargement of the ........ 257
Kidney, suppuration of the ........ 262

Kiestein in the urine of pregnant women ........................ 229
Kirkbride on wounds of arteries .... 184
Kluge's treatment of syphilis ...... 286
Knee-joint, Merryon on diseases of the 299

L.

Laceration of the perinæum cured .. 605

Lacerations of the recto-vaginal septum 529

Lacerations of the perinæum, M. Roux on ........................ 526

Last illness of Broussais ........... 197

Lateral deviations of the spine ...... 523

Lateral curvature, operation for .... 575

Leeches, mode of breeding them in the Deccan .................. 390

Legroux on endocarditis ........... 209

Lendrick on incontinence of urine .. 567

Letter from Mr. Fiddes ............ 305

Life of Dr. Willis ................ 347

Life, influence of physical agents on.. 385

Lindley's school botany............ 179

Lisfranc's treatment of amaurosis ... 219

Liston on poultices................ 282

Literary and scientific medical men .. 343

Lithic acid gravel, mode of giving alkalies for ...................... 38

Living animals, Turner's experiments on ......................... 505

Living vessels, condition of the blood in the ....................... 507

Locke, anecdotes of ............... 345

Lombard on typhus fever .......... 541

Longevity in different counties...... 446

Louis as a practical man............ 542

Lues venerea ...................... 483

M.

M'Cabe on the treatment in iritis.... 597
Macfarlane's, Dr., case ............ 601
Macreight's manual of British botany 179
Mad-doctors and mad-houses ...... 347
Malignant ulcers of the tongue...... 383
Malignant ulcers of the tonsils..... 383
Mallet on hernia cerebri........... 45
Malpraxis of M. Dubois............ 548
Malta, medical statistics of ........ 16
Mamma, inflammation of the ....... 382
Mammæ, case of cancer of the... .. 236
Manual for students, Meade's ....... 177
Marc on insanity.................. 206
Marjolin on scrofula and syphilis .... 213
Marsh miasma and fever............ 477
Massachusett's hospital, report of the, 263
Meade's manual for students........ 177
Mean annual mortality ............ 458
Mechanism of blushing ............ 173
Mechanism of the bruit de soufflet .. 575
Medical notes and reflections, by Dr. Holland ....................... 81
Medical portrait gallery............ 122
Medical and physical researches, Harlan's........................... 149
Medical valets...................... 287
Medical Portrait Gallery ........... 309
Medical Sketch-book .............. 309
Medical quackery, illustrations of.... 317
Medical men, literary and scientific .. 343
Medical charities, abuses and evils of, 357
Medical notes and reflections, by Dr. Holland ........................ 392
Medical science near the Indus...... 387
Medical treatment of old age........ 400
Medical evidence .................. 409
Medical statistics ................. 443
Medical practitioners, circular to.... 448
Medical memoranda ............... 501
Medical quackery, Dr. Cowan on.... 511
Medicine, Elliotson's practice of .... 366
Medicine, progress of, in America.... 584
Medicines, observations on ........ 84
Mediterranean, sickness of troops in 11
Melituria.......................... 34
Membrana tympani, tumefaction of the 281
Menorrhagic chlorosis ............ 223
Mental attention, effects of, on the bodily organs .................. 181
Mental emotions, deaths from ...... 454
Mercurial medicines .............. 88
Mercurial rheumatism ............ 483
Mercury in syphilis, Dr. Murphy on.. 479
Mercury, administration of ........ 484
Merryon on diseases of the knee-joint, 299
Microscope, novel application of the.. 203
Midwifery in private practice, Mr. Rose on ...................... 42
Midwifery, Dr. Blundell on........... 177
Military medical reports ........... 1
Military medical statistics.......... 443
Milky urine........................ 26
Mode of giving alkalies for lithic acid gravel ........................ 38
Mode of administering iron ..... .. 225
Mode of exhibiting iodine .......... 378
Mollities ossium, Dr. Rees on ...... 246
Molluscum, case of, by Mr. Fowler .. 47
Monsey, anecdotes of............... 320
Montrose Lunatic Asylum .......... 591
Morbid changes of typhus.......... 117
Morbid growths, hypotheses of...... 355
Morgan on the diseases of the eye.... 64
Morphia, Squire's bimeconate of .... 190
Mortality among officers ........... 9
Mortality from influenza .......... 452
Mortality of troops in British America 460
Mortality in North America ........ 475
Motor roots, sensibility of the ...... 577
Mouth, gangrenous inflammation of, 560
Mucous membrane, M. Flourens on.. 522
Mucus of the urine ............... 31
Murphy on mercury in syphilis...... 479
Murray, Sir J. his reclamation ...... 598
Murray's, Sir J. fluid magnesia...... 598

N.
Nævi, treatment of, with caustic .... 227
Nasmyth on the teeth .............. 138
Nervous system, comparative anatomy of the .................... 178
Neuralgia, intermittent, hints on .... 545
New mode of treating prolapsus uteri, 285
Newfoundland, climate of .......... 473
Nitrate of silver, application of, to the eyelids ....................... 75
Nitrate of silver for inflammation of the prostate .................. 580
Nodes ........................... 483
Nova Scotia, diseases of........... 462
Novel application of the microscope.. 203
Nux vomica, utility of, in paralysis .. 217
O.
Observations on various medicines .. 84
Observations on vaccination........ 555
Obstetrical advice ................ 548
Obstructed nasal duct, treatment of.. 78
Obstruction of the bowels...... ... 576
Obstructions of the rectum ........ 233
Occlusion of the cervix uteri ....... 254
Occlusion of the vagina and uterus .. 571
Old age, medical treatment of ...... 400
Oleo-albuminous urine ............ 25
Ophthalmia, catarrhal ............ 66
Ophthalmia from extraneous bodies.. 66
Ophthalmia, variolous ............ 71
Ophthalmia, pustular.............. 72
Ophthalmia of purulent infants ..... 73
Ophthalmia, strumous ............ 74
Ophthalmia, applications in ........ 273
Ophthalmia, scrofulous ............ 274
Ophthalmia, scrofulous, treatment of, 540
Ophthalmia treated by blistering the eyelids ....................... 562
Operation for lateral curvature...... 575
Opiates, use of .................. 89
Opium as a diaphoretic ............ 85
Orchitis ......................... 215
Orfila on the detection of the salts of lead ......................... 208
Osborn on dropsies ................ 295
Os uteri, Dr. Kennedy on hypertrophy of the ....................... 159
Os uteri, dilatation of the, in lingering labour .......................... 43
Ozœna, treatment of .............. 561
P.
Paralysis, bleeding in.............. 82
Paralysis, utility of nux vomica in.... 217
Paralysis of the arm, case of........ 220
Paralysis of the bladder, secale cornutum in ....................... 221
Paralysis, ergot of rye in .......... 222
Paraplegia, secale cornutum in...... 546
Parsley in gonorrhœa.............. 221
Partial sweating, case of........... 566
Patency of the foramen ovale ...... 211
Pathology and surgery, selections in, 377
Pennsylvania Hospital, report of the, 268
Perinæum, Roux on lacerations of the 526
Pettigrew's Medical Portrait Gallery, 122
Pettigrew's Medical Portrait Gallery, 309
Philip on the vital function ........ 177
Phlegmon ....................... 380
Phthisis, Dr. Furnival on .......... 289
Physic and physicians ............ 309
Physic, art of rising in ............ 310
Physiology, Mr. Carpenter on ...... 165
Physiology of blushing ........... 173
Pins and needles, case of swallowing.. 205
Pitting in small-pox, prevention of .. 574
Platina wire for uniting wounds .... 579
Poetry of blushing ............... 173
Points where a patient may judge for himself ....................... 421
Poison of the rattle-snake, experiments on the ....................... 155
Poisoning by extract of aconite...... 544
Pommade for baldness, Dupuytren's.. 240
Popliteal tendons, spasmodic contraction of the .................. 526
Porrigo, Dr. Dick on.............. 502
Porrigo lupinosa.................. 503
Porrigo, treatment of.............. 504
Poultices, Mr. Liston on ... ....... 282
Powers and applications of iodine.... 377
Practice, how to get a............. 310
Practitioners at home and abroad. .. 288
Pregnancy, duration of ............ 424
Pregnant women, kiestein in the urine of ....................... 228
Prescribing, modes of.............. 419
Prevalence of suicide among the military ........ ................. 4
Prevention of pitting in small-pox ... 574
Principles and practice of medicine, Elliotson's ................... 366
Progress of surgical literature ...... 578
Prolapsus ani ................... 267
Prolapsus uteri, treatment of........ 285
Prolific uterus, with barren mammæ, case of............... ....... 150
Prospects of vaccination .......... 397
Provincial Medical and Surgical Association, transactions of the ... .... 40
Proximate cause of diabetes ........ 35
Psoas abscess, dependence of, on diseased bone .................. 278
Pterygium pinque ................ 75
Ptisane de Feltz ..... .......... 603
Pulmonary artery, aneurysm of the .. 149
Pulmonary affections ............ 476
Pulse, Guy on the diurnal variations of the ...................... 244
Purgative medicines, abuse of . .... 86
Purkinge's observations on the teeth, 141
Purpura hæmorrhagica ........... 305
Purulent urine .................. 32

Purulent ophthalmia of infants...... 73
Pus globule, description of the ...... 352
Pustular inflammation of the conjunctiva ........................ 68
Q.
Quackery, medical, illustrations of .. 317
Quackery, medical, Dr. Cowan on .. 511
R.
Radius, dislocation of the upper end of the ........................ 580
Rash of typhus fever ............... 100
Rattlesnake, experiments on the poison of the ........................ 155
Rayer sur les maladies des reins .... 25
Recto-vaginal septum, lacerations of, 529
Rectum, obstructions of the ........ 233
Rectum, fissure of the ............ 264
Rees on mollities ossium .......... 246
Registrar general, annual report of the 443
Registration, success of the new .... 443
Registration of births.............. 445
Relations of food and sleep ....... 415
Remarkable case of partial sweating.. 566
Report of the Dublin Cow-pox Institution ........................ 188
Report of the Massachusett's hospital 263
Respiratory apparatus, M. Serres on the ........................ 520
Retention of urine, use of strychnia in 191
Retzius' observations on the teeth .. 143
Re-vaccination, Dezeimeris on ...... 193
Re-vaccination ................... 398
Re-vaccination ................... 578
Rheumatism ...................... 477
Rheumatism of the uterus.......... 563
Ricord on syphilitic condyloma...... 215
Ricord on iodide of potassium in syphilis ........................ 539
Ringworm ........................ 503
Roberton on vaccination .......... 170
Rose on midwifery in private practice, 42
Roupell on typhus fever .......... 96
Roux on lacerations of the perinæum, 526
Rupture of the spleen, fatal ........ 391
Ruptures of the heart, M. Dezeimeris on ........................ 531
Ruptures of the heart, spontaneous.. 532
S.
Salts of lead, Orfila on the detection of 208
Scrofula, M. Marjolin on .......... 213
Scrofulous ophthalmia ............ 274
Scrofulous disease of the hip-joint ... 279
Scrofulous ophthalmia, treatment of, 540
Scudamore on the springs of Buxton, 162
Secale cornutum in paralysis of the bladder ........................ 221
Secale cornutum in paraplegia ...... 546
Section of the tendo-achillis........ 201
Selections in pathology and surgery.. 377
Sensibility of the motor roots ...... 577
Serres on the respiratory apparatus .. 520
Shakspeare, cause of his death ...... 501
Sheffield, state of society in ........ 357
Sickness of troops in British America, 460
Signs of death from hanging........ 550
Size of varicose capillaries.......... 354
Sketches of medical quackery ...... 317
Sleep and food, relations of ........ 415
Small-pox, deaths from............ 453
Small-pox, prevention of pitting in.. 574
Society in Sheffield, state of........ 357
Spasmodic contraction of the popliteal tendons ........................ 526
Speculum, Jones on the use of the ... 53
Spider, bite of a, on the glans penis.. 565
Spine, lateral deviations of the...... 523
Spine, operation for lateral curvature of the ........................ 575
Spleen, tubercle of the ............ 355
Spleen, fatal rupture of the ........ 391
Spontaneous rupture of the heart.... 532
Springs of Buxton, Sir C. Scudamore on the ........................ 162
Squire's bimeconate of morphia .... 190
Statistics, military medical ....... 1
Stomach, capillaries of the ......... 355
Stomatitis, treatment of............ 560
Structure of the teeth ............ 141
Strumous ophthalmia .............. 74
Strychnia in retention of urine...... 191
Students, Meade's manual for ...... 177
Studentships in anatomy .......... 284
Studies of an Indian physician ...... 389
Styptic action of creosote .......... 225
Success of the new system of registration ........................ 443
Sudorific medicines ............... 85
Sugar of lead in dysentery ......... 150
Suicide, Whitfield on the causes of .. 282
Suppuration of the kidney.......... 262
Surgery and pathology, selections in.. 377
Surgical literature, progress of ...... 578
Surgical injuries, Mr. Alcock on .... 488
Swallowing of pins and needles, cases of 205
Swallowing pins and needles........ 288
Swan's plates ..................... 178
Syphilis, M. Marjolin on .......... 213
Syphilis and gonorrhœa............ 214
Syphilis, compound salts of iodine and mercury in ........................ 216
Syphilis, Kluge's treatment of ...... 286
Syphilis, iodide of potassium in...... 539
Syphilis, Dr. Murphy on mercury in.. 479
Syphilitic iritis, diagnostic marks of.. 76
Syphilitic condyloma, M. Ricord on.. 215
T.
Talma, death of ................... 233
Tartar emetic, utility of, in large doses 211
Teeth, structure of the ............ 141
Teeth, Mr. Nasmyth on the ........ 138
Teeth, Mr. Wardroper on the ...... 502
Tegumentary system, diseases of the 454
Tendo-achillis, section of the ...... 201
Tetanus ........................ 374

Theory of life.................... 169
Therapeutical memoranda ........ .. 221
Thigh, fractures of the ............ 263
Thompson on a peculiar affection of the uvula ......................... 41
Throat, ulceration of the............ 482
Throat, venereal ulceration of the.... 482
Tibia, division of the.............. 249
Tongue, cancer of the............ 267
Tongue, malignant ulcers of the .... 383
Tonsils, malignant ulcers of the .... 383
Tophi ........................ 483
Towns, diseases of ................ 455
Traité des maladies des reins. Par P. Rayer ...................... 25
Transactions of the Provincial Medical and Surgical Association ...... . 40
Transactions of the medical society of Bombay ....................... 384
Traumatic paralysis of the arm, Blandin on ........................ 220
Traumatic delirium, frequency of.... 269
Treatment of diabetes ............ 36
Treatment of strumous ophthalmia .. 74
Treatment of scrofulous ophthalmia.. 540
Treatment of stomatitis............ 560
Treatment of delirium tremens...... 572
Trousseau's treatment of menorrhagic chlorosis ...................... 223
Tubercle of the spleen ............ 355
Tulloch's military medical reports ... 1
Tumefaction of the membrana tympani ......................... 281
Turner's experiments.............. 505
Turpentine, internal administration of 226
Turpentine for iritis ........... .. 280
Tweedie on imperforate uterus...... 251
Tympanitis ................ ..... 406
Typhus fever, Dr. Roupell on ...... 96
Typhus fever, definitions of ........ 99
Typhus, rash of .................. 100
Typhus, contagious nature of ...... 104
Typhus, causes of ............... 106
Typhus, complications of .......... 109
Typhus, morbid changes of ........ 117
Typhus fever, M. Lombard on ...... 541

U.

Ulceration of the throat............ 482
Ulcers .......................... 383
United Kingdom, sickness of troops in 1
University College hospital ........ 276
Upper Canada, diseases of.... ..... 468
Urea, existence of, in diabetic urine.. 34
Urethra, vascular tumor of the ...... 587
Uric acid, decomposition of iodic acid by.... ...................... 574
Urinary organs, diseases of the.... . 25
Urinary diseases, Dr. Willis on...... 25
Urinary calculi, formation and growth of .......................... 38
Urinary organs, disease of the ...... 454
Urine, mucus of the .............. 31
Urine having the elements of the blood mixed with it .................. 28
Urine, purulent .................. 32
Urine, strychnia for retention of .... 191
Urine of pregnant women.......... 229
Urine, Dr. Lendrick on incontinence of 567
Use of diluents .................. 85
Uterus, Ashwell on incision of the .. 253
Uterus, rheumatism of the ........ 563
Uterus, Dr. Kennedy on occlusion of 571
Uterus, absence of the ............ 546
Utility of tartar emetic in large doses 211
Utility of nux vomica in paralysis .. 217
Utility of belladonna in epilepsy .... 543
Uvula, Mr. Thompson on a peculiar affection of the ................ 41

V.

Vaccination, prospects of .......... 397
Vaccination, Mr. Roberton on ...... 170
Vaccination in Barataria............ 171
Vaccination, antiquity of .......... 286
Vaccination, observations on........ 555
Vagina, occlusion of the .......... 570
Vagina, occlusion of the, means of preventing .................... 571
Varicocele, Cooper's treatment of.... 247
Varicocele, successful treatment of .. 270
Varicose capillaries the cause of carcinoma........................ 351
Varicose capillaries, size of the...... 354
Varicose capillaries, rupture of...... 355
Variolous inflammation of the conjunctiva ...................... 70
Variolous ophthalmia.............. 71
Vegetable organography, by De Candolle............................ 179
Venereal ulceration ................ 275
Venereal affections of the bones .... 483
Venous pulsation, Allison's experiments on ...................... 189
Vertebral diseases, Sir B. C. Brodie on 277
Vertigo ........................ 82
Vital principle.................... 167
Vital function, Dr. Philip on the .... 177

W.

Warburg's fever drops ............ 50
Ward, the Rev. Mr., his diary ...... 501
Wardroper on the teeth............ 502
Waterton on the wourali poison .... 185
Weather, influence of the .......... 393
West Indies, Sir A. Halliday on the.. 49
Western Lying-in Hospital, report of 272
Whitfield on the causes of suicide.... 282
Whitlow .................... .... 383
Willis on urinary diseases .......... 25
Willis, life of .................... 347
Wolff on delirium tremens.......... 492
Women, Jones on the diseases of .... 53
Wourali poison, Waterton on the.... 185

Y.

Yarrell's history of British birds .... 179
Yellow-fever in Gibraltar .......... 12

### BIBLIOGRAPHICAL RECORD.

1. School Botany; or an Explanation of the Character and Differences of the principal natural Classes and Orders of Plants belonging to the Flora of Europe, in the Botanical Classification of De Candolle. For the Use of Students preparing for their Matriculation Examination in the University of London. By John Lindley, Ph. D. F.R.S. Small 8vo, pp. 218, with numerous woodcuts, and a copious Index. Longman and Co. 1839.

2. Hints to Mothers, &c. By Thomas Bull, M.D. Physician-Accoucheur to the Finsbury Midwifery Institution. Octavo, pp. 307. Second Edition, much enlarged and improved. Longman and Co. 1839.

3. A Letter to the Right Honorable the Secretary at War, on Sickness and Mortality in the West Indies; being a Review of Capt. Tulloch's Statistical Report. By Sir Andrew Halliday, M.D. F.R.S. &c. Deputy Inspector of Army Hospitals. 8vo. pp. 63. J. W. Parker, West Strand. March, 1839.

4. A short Treatise on Typhus Fever. By George Leith Roupel, M.D. Physician to St. Bartholomew's Hospital, &c. 8vo. pp. 301. London, 1839.

5. The Phrenological Journal, and Magazine of Moral Science, No. 59. April 1839. Edited by Hewett Cottrill Watson, F.L.S. Simpkin and Marshall.

6. Lectures on Diseases of the Eye. By John Morgan, F.L.S. Surgeon to Guy's Hospital, and Lecturer on Surgery at that Institution. Illustrated by coloured Plates. Octavo, pp. 221. Highley, 1839.

7. An Inquiry into the Propagation of Contagious Poisons by the Atmosphere; and also into the Nature and Effects of Vitiated Air, its Forms and Sources, and other Causes of Pestilence; with Directions for avoiding the Action of Contagion, and Observations on some Means of promoting Public Health. By S. Scott Alison, M.D. Octavo. pp. 219. Maclachlan, Ed. Whittaker, London. April, 1839.

8. Statistical Reports on the Sickness, Mortality, and invaliding among the Troops of the United Kingdom, the Mediterranean, and British America; prepared from the Records of the Army Medical Department, and War Office Returns. Folio, 1839.—"Presented to Dr. James Johnson by Sir James M'Grigor."

---

9. The Physiognomy of Mental Diseases. By Sir ALEX. MORISON, M.D. No. 10, Monomania, with Vicious Propensities. London, 1839.

---

10. Medical Notes and Reflections. By HENRY HOLLAND, M.D. &c. Octavo, pp. 628. Longman and Co. 1839.

---

11. A practical Compendium of the Materia Medica, adapted to the Diseases of Infancy and Childhood, with numerous Prescriptions. By ALEX. URE, M.D. &c. An enlarged Edition, containing simple Rules for the Diet and Regimen of Children, &c. Duodecimo, 1839. Sherwood and Co.

---

12. An Experimental Inquiry into the Laws of the Vital Functions, &c. &c. By A.P.W. PHILIP, M.D. Fourth Edition, greatly enlarged, both in the physiological and practical part. Renshaw, London, 1839.

---

13. A Series of Anatomical Sketches and Diagrams, with Descriptions and References. By THOMAS WORMALD, Assistant Surgeon, &c. Bartholomew's Hospital, and A. M. M'WHINNIE, Teacher of Practical Anatomy, &c. Quarto, Part II. Highley, 1839.

---

14. Mind and the Emotions considered in Relation to Health and Disease. By W. COOKE, M.D. Editor of Morgagni, &c. Octavo, pp. 56. (An Oration.) Longman and Co. 1839.

---

15. Total Abolition of personal Restraint in the Treatment of the Insane. With Statistical Tables, &c. By ROBERT GARDINER HILL, M.R.C.S. House-Surgeon of the Lincoln Lunatic Asylum. Octavo. Simpkin and Co. London, 1839.

---

16. Dr. Milner's Address to the Candidates for Degrees and Licences in the Medical Institution of Yale College, United States.

17. Dr. J. Knight's Introductory Lecture to the Course of Instruction in Yale College. New Haven, 1838.

---

18. Professor Pattison's Introductory Lecture, Session 1838—9, Philadelphia, 1839.

---

19. Outlines of Human Physiology. By W. P. ALISON, M.D. F.R.S. Third Edition. Blackwood, Edinb. 1839.

*** *In this Edition the talented Author has introduced all the most important facts which have been lately ascertained in this department of Science. We need hardly recommend a work which has so well recommended itself.*

---

20. Reflexions sur les Œuvres de Shakespeare. Hommage a la Societé d'Emulation pour les Lettres et les Arts a Paris. Par W. PATTISON HUNTER. Jan. 1839.

*** *We beg to return Mr. Hunter our thanks for this interesting Essay.*

---

21. Observations on "Oxygenous Ærated Water," as a grateful exhilarating Beverage, and as a Remedy in Debility, Depression of Spirits, &c. By C. SEARLE, Esq. M.R.C.S.L. late of the Madras Establishment. London, 1839.

---

22. Mr. James Law's Address at the First Anniversary Meeting of the Birmingham School of Medicine and Surgery.

*** *Clerical and pious.*

---

23. Report of the Royal School of Medicine and Surgery, Birmingham. 1838.

---

24. An Address delivered at the Birmingham Royal School of Medicine and Surgery. By VAUGHAN THOMAS, B.D.

---

25. The Warneford Prize Essay, (Birmingham School of Medicine,) on the Valvular of the Veins, anatomically and physically considered, &c. By THOMAS CLARKE RODEN, Student. 1839.

---

26. An Inquiry into the local, moral, and intellectual Condition of the Industrious Classes of Sheffield. Part I. The Abuses and Evils of Charity, especially of Medical Charitable Institutions. Royal 8vo, pp. 132. Longmans, 1839.

---

27. Case of Hydrophobia in Nottingham. By J.C. WILLIAMS, M.D.

*** *We fear this case will not throw any light on the invariably fatal nature of the disease, nor indicate any remedy.*

28. Experiments with the Wourali Poison. By Mr. WATERTON.

29. Answers to the Objections commonly brought against Vaccination. By J. ROBERTON, Esq.

30. Extracts from a Report made to the New York and Maryland Iron Company, &c. By the Messrs. SILLIMANS. London, 1838.

31. Researches on the Development, Structure, and Diseases of the Teeth. By ALEX. NASMYTH, F.L.S. Octavo, pp. 165, with Plates. Churchill, London, May 1839.

32. Elements of the Practice of Medicine. By Drs. BRIGHT and ADDISON. Part III. Price 7s. Longman and Co. June, 1839.

33. The Accoucheur; a Treatise on protracted Natural Labours, suspended Animation in New-born Infants, and Uterine Hæmorrhage after the Birth of the Child; with illustrative Cases. By JOHN CRAIG, Surgeon. Paisley. Octavo, pp. 252. W. R. M'Phun, 1839.

34. The Transactions of the Provincial Medical and Surgical Association. Instituted 1832. Vol. VII. Churchill, London, June, 1839.

*** *We observe that the next anniversary meeting of the Association will be held at Liverpool, on the 24th and 25th of July of this year.*

35. Elements of Physiology. By J. MULLER, M.D. Translated from the German, with Notes, by W. BALY, M.D. Illustrated with steel plates and numerous wood-engravings. Part V. containing the Senses. (Taylor and Walton, London, June, 1839.) Price 4s. 6d.

36. Sleep and its Phenomena. An Essay. By JAMES N. PINKERTON, M.D. Small 8vo. pp. 220. Fry and Son, London, June 1839.

37. Outlines of Pathological Semeiology. Translated from the German of Professor Schill. With copious Notes. By D. SPILLAN, M.D. Small 8vo, pp. 220. Renshaw, 1839.

*** *We lately gave a Review of the original Work in this Journal, and think the Profession is indebted to Dr. Spillan for a translation in an economical form.*

38. A General Outline of the Animal Kingdom, and Manual of Comparative Anatomy. By T. RYMER JONES, F.Z.S. Part VI. June 1, 1839. Price 2s. 6d.

39. Supplement to the History of British Fishes. By WILLIAM YARRELL, F.L.S. Illustrated with Wood-cuts. In two parts. Van Voorst, Paternoster-row, 1839.

40. Practical Remarks on the Use of Iodine, locally applied in various Surgical Diseases and External Injuries. Illustrated by Cases. By JOHN DAVIES, Surgeon to the General Infirmary at Hertford, &c. Octavo. Longmans & Co. June 1839.

41. Selections in Pathology and Surgery; or, an Exposition of the Nature and Treatment of Local Disease; exhibiting new Pathological Views, and pointing out an important practical Improvement. Illustrated by Cases. By JOHN DAVIES, Surgeon to the General Infirmary at Hertford, &c. Octavo. Longmans & Co. June 1839.

☞ *Several Works have been received for review since the above list was closed.*

